# Polymorphisms in promoter sequences of *MDM2, p53*, and *p16*^*INK4a*^ genes in normal Japanese individuals

**DOI:** 10.1590/s1415-47572010000400004

**Published:** 2010-12-01

**Authors:** Yasuhito Ohsaka, Hoyoku Nishino

**Affiliations:** 1 Department of Biochemistry and Molecular Biology, Graduate School of Medical Science, Kyoto Prefectural University of Medicine, KyotoJapan; 2 Department of Pharmacology, Faculty of Pharmaceutical Sciences, Chiba Institute of Science, ChibaJapan; 3 Ritsumeikan Global Innovation Research Organization, Ritsumeikan University ShigaJapan

**Keywords:** Murine double minute 2, polymorphism, p16^INK4a^, p53, transcription

## Abstract

Research has been conducted to identify sequence polymorphisms of gene promoter regions in patients and control subjects, including normal individuals, and to determine the influence of these polymorphisms on transcriptional regulation in cells that express wild-type or mutant p53. In this study we isolated genomic DNA from whole blood of healthy Japanese individuals and sequenced the promoter regions of the *MDM2*, *p53*, and *p16*^*INK4a*^ genes. We identified polymorphisms comprising 3 nucleotide substitutions at exon 1 and intron 1 regions of the *MDM2* gene and 1 nucleotide insertion at a poly(C) nucleotide position in the *p53* gene. The Japanese individuals also exhibited *p16*^*INK4a*^ polymorphisms at several positions, including position -191. Reporter gene analysis by using luciferase revealed that the polymorphisms of *MDM2*, *p53*, and *p16*^*INK4a*^ differentially altered luciferase activities in several cell lines, including the Colo320DM, U251, and T98G cell lines expressing mutant p53. Our results indicate that the promoter sequences of these genes differ among normal Japanese individuals and that polymorphisms can alter gene transcription activity.

## Introduction

Different populations exhibit sequence polymorphisms of the murine double minute 2 (*MDM2*) gene (Atwal *et al.*, 2007), a target gene of the transcription factor p53. A promoter polymorphism of the *MDM2* gene, SNP309, is located in the intron 1 region of the gene and influences transcriptional regulation in a cell line expressing wild-type p53 (Bond *et al.*, 2004). Non-cancerous control subjects frequently have a polymorphism of p53 at codon 72 (Wu *et al.*, 1995; Minaguchi *et al.*, 1998), and this common polymorphism differentially alters promoter activity of the *MDM2* gene that contains the SNP309 polymorphism (Yang *et al.*, 2007). Cytosine-phospho-guanine (CpG) dinucleotides are methylated by DNA methyltransferase (DNMT) (Siedlecki and Zielenkiewicz, 2006), and the methylated form interacts with methyl-CpG-binding proteins (Fan and Hutnick, 2005), which serve as modulators of gene transcription. CpG methylation in the promoter region of *p53* decreases promoter activity of the gene (Schroeder and Mass, 1997). Genomic DNA obtained from blood of people exposed to arsenic has been reported to exhibit methylation at the promoter regions of *p53* and *p16*^*INK4a*^, a cycline-dependent kinase inhibitor, and the DNA methylation status of these promoters differs among people (Chanda *et al.*, 2006). Normal populations have polymorphisms in *DNMT*; a polymorphism *DNMT3L* affects the ability of this gene to stimulate DNA methylation (El-Maarri *et al.*, 2009). Nucleotide substitutions in the *p53* promoter at 4 mutated positions, including position -250, and in the *p16*^*INK4a*^ promoter at positions -735, -493, and -191 are found not only in Taiwanese patients with uterine leiomyoma (Hsieh *et al.*, 2007) and frequently in melanoma families from other populations (Harland *et al.*, 2000) but also in control groups; *p53* promoter polymorphism at position -250 and *p16*^*INK4a*^ promoter polymorphism at position -191 are located within CpG dinucleotides. Thus, control subjects, including normal populations, exhibit gene promoter polymorphisms. However, no studies have investigated differences at other nucleotide positions in *MDM2*, *p53*, and *p16*^*INK4a*^ promoter sequences among healthy individuals. In addition, it has not been determined whether known polymorphisms in the promoter sequence of these genes are present in other normal populations and alter gene promoter activity in other cell lines.

A number of polymorphisms have been identified in 10 ENCODE (Encyclopedia of DNA Elements) regions in the human genome of 48 individuals from 4 populations, including 8 Japanese individuals. Moreover, the frequency distributions of these polymorphisms have been investigated among different populations (International HapMap Consortium, 2005). In the present study, we sequenced the *MDM2* and *p53* promoters and the *p16*^*INK4a*^ promoter nucleotides at positions -735, -493, and -191 from genomic DNA extracted from whole blood samples obtained from healthy Japanese individuals and determined whether these promoter polymorphisms affect gene promoter activity in cell lines expressing mutant or wild-type p53. We found that normal Japanese individuals exhibit polymorphisms in these regions of the *MDM2*, *p53*, and *p16*^*INK4a*^ genes and that these polymorphisms alter promoter activity in some cell lines.

## Materials and Methods

###  Extraction of genomic DNA

Human peripheral blood was obtained from 17 healthy Japanese students, who consented to have their DNA sequenced for identification of polymorphisms. Genomic DNA was extracted from whole blood by using a QIAamp DNA Blood Mini kit (Qiagen, Hilden, Germany). DNA analysis of the samples showed that the frequency of commonly known polymorphisms (Kadowaki *et al.*, 1995; Eguchi-Ishimae *et al.*, 2005) was similar to that previously identified in control Japanese subjects.

###  Amplification of promoter regions by polymerase chain reaction

Promoter sequences of the *MDM2* genes were amplified by polymerase chain reaction (PCR) in a reaction mixture containing genomic DNA (0.1 μg) and MDM2 primers (Table 1) in the presence or absence of 5% dimethyl sulfoxide with DNA polymerase (KOD-Plus DNA polymerase (Takagi *et al.*, 1997); Toyobo, Osaka, Japan) according to the manufacturer's instructions. The amplification was performed in a thermal cycler (Takara PCR thermal cycler MP; Takara, Osaka, Japan) under the following conditions: denaturation at 94 °C for 15 s, annealing at (melting temperature [Tm]-5) °C for 30 s, and extension at 68 °C for 2 min. *p53* and *p16*^*INK4a*^ promoter sequences were amplified using p53 and p16^INK4a^ primers (Table 1) and DNA polymerase (PfuTurbo DNA polymerase (Cline *et al.*, 1996); Stratagene, La Jolla, CA) according to the manufacturer's instructions under the following conditions: denaturation at 94 °C for 30 s, annealing at (Tm-5) °C for 1 min, and extension at 72 °C for 2 min.

###  Determination of nucleotide sequences

Sequence reactions were performed using an ABI PRISM Dye Terminator Cycle Sequencing kit (Perkin-Elmer Biosystems, Foster City, CA), and nucleotide sequences were determined using the ABI PRISM 377 automated DNA sequencer (Perkin-Elmer Biosystems).

###  Construction of reporter plasmid vectors

Regions from positions -725 to +99 of the *MDM2* gene, positions -920 to +184 of the *p53* gene, and positions -1703 to -93 of the *p16*^*INK4a*^ gene were amplified by PCR with primers bearing a cleavage site of the restriction enzyme *KpnI* or *BglII*. The amplified fragments were digested and separated by agarose gel electrophoresis. DNA fragments were extracted using a gel extraction kit (Concert Gel Extraction System; Invitrogen, Carlsbad, CA), precipitated with ethanol, and ligated between the *KpnI* and *BglII* sites of the PicaGene Basic Vector 2 (PGV-B2) reporter plasmid (Nippon Gene, Tokyo, Japan). The constructed vectors were cloned in *Escherichia coli* DH5α (Toyobo) and then purified using a QIAfilter Plasmid Midi kit (Qiagen). Nucleotide sequences were confirmed using primers (Table 1), including the PGV-B2 vector primers.

###  Analysis of nucleotide sequences and polymorphisms

The nucleotide sequences obtained for 17 individuals (or 9 individuals in the case of *p16*^*INK4a*^) were compared with DNA sequences (accession numbers U39736.1, U28935.1, X54156.1, J04238.1, M13111.1, U94788.1, AF022809, and X94154.1) in the DNA database GenBank at the National Center for Biotechnology Information by using the program BLAST (basic local alignment search tool). For detection of polymorphisms, nucleotides were compared among individuals by using the sequence alignment editor program BioEdit.

###  Cell culture

Human colon adenocarcinoma Colo320DM (American Type Culture Collection [ATCC], Manassas, VA) and breast carcinoma YMB-1 (Japanese Collection of Research Bioresources [JCRB], Osaka, Japan) cell lines were grown in RPMI 1640 medium (Nissui Pharmaceutical, Tokyo, Japan). The human glioma U251 (Riken Cell Bank [RCB], Tsukuba, Japan) cell line was grown in Dulbecco's modified Eagle's medium (Nissui Pharmaceutical). Human glioblastoma T98G and cervix epithelioid carcinoma HeLa cell lines (RCB) were cultured in Eagle's minimum essential medium (Nissui Pharmaceutical) containing nonessential amino acids and sodium pyruvate (Invitrogen). These cells were grown at 37 °C in media supplemented with 10% fetal bovine serum (Trace Scientific, Melbourne, Australia) in a humidified atmosphere of 95% air and 5% CO_2_.

###  Transfection and reporter gene analysis

The cultured cells were plated at a density of 1.04 x 10^4^ cells/cm^2^ and then transfected with 1 μg of vector DNA using Lipofectamine 2000 (Invitrogen) according to the manufacturer's instructions. The transfected cells were cultured and then lysed after 24 h in a reporter lysis buffer (Promega, Madison, WI). Luciferase was used as a reporter gene, and luciferase activity was determined using the Luciferase Assay System (Promega) in a luminometer (Monolight 2010; Analytical Luminescence Laboratory, San Diego, CA). For correction of variations in the amount of vector DNA incorporated into cells, the cultured cells were co-transfected with pCMVβ (Clontech, Palo Alto, CA), a β-galactosidase expression vector. Luciferase activity was normalized according to the β-galactosidase activity.

###  Statistical analysis

Comparisons among multiple groups were performed using analysis of variance (ANOVA) with a *post hoc* test. Differences between two groups were evaluated using unpaired Student's *t* test. Statistical significance was defined as p < 0.01.

## Results

###  Polymorphisms in promoter regions of the *MDM2*, *p53*, and *p16*^*INK4a*^ genes

**Figure 1:**
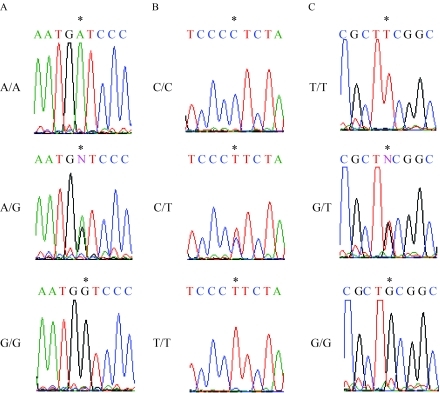
Promoter polymorphisms of *MDM2* at positions -628, -466, and -215. (A-C) Genomic DNA was amplified by PCR with a forward primer at positions -725 to -704 and a reverse primer at +96 to +75 of the *MDM2* gene, and nucleotide sequences were determined by directly sequencing the PCR products. These sequencing reactions were performed using primers at positions -725 to -704 and -310 to -289. (*) shows nucleotides at positions -628 (A), -466 (B), and -215 (C) in the *MDM2* promoters, where “N” indicates heterozygous nucleotides. Nucleotides around these positions (*) are indicated.

**Figure 2:**
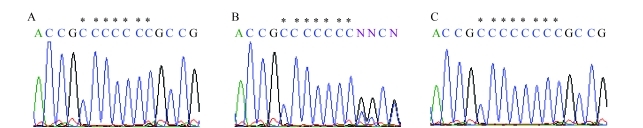
A poly(C) polymorphism of *p53* at positions -824 to -818. (A-C) Genomic DNA was amplified by PCR with a forward primer at positions -918 to -899 and a reverse primer at -669 to -690 of the *p53* gene. Nucleotide sequences were determined by directly sequencing the PCR products; these sequencing reactions were performed using a primer at positions -918 to -899. (*) shows homozygous (A and C) or heterozygous (B) nucleotides at positions -824 to -818 in the *p53* promoters, where “NNCN” in (B) indicates sequences including “GCCG” and “CGCC,” which result from nucleotide C insertion. Nucleotides around the poly(C) polymorphic position (*) are indicated.

**Figure 3:**
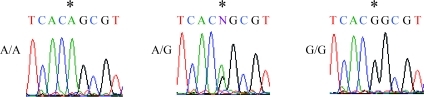
A polymorphism of *p16*^*INK4a*^ at position -191. Genomic DNA was amplified by PCR with a forward primer at positions -485 to -465 and a reverse primer at +214 to +194 of the *p16*^*INK4a*^ gene, and nucleotide sequences were determined by directly sequencing the PCR products. These sequencing reactions were performed using a primer at positions -248 to -228. (*) shows nucleotides at position -191 in the *p16*^*INK4a*^ promoters, where “N” indicates heterozygous nucleotides. Nucleotides around the polymorphic position (*) are indicated.

We amplified the regions containing *MDM2* and *p53* promoters (Tuck and Crawford, 1989; Zauberman *et al.*, 1995) in genomic DNA isolated from 17 healthy individuals by PCR and determined the nucleotide sequences of the amplified regions. Comparisons of the nucleotide sequences among individuals revealed that nucleotide A was substituted with nucleotide G at position -628 in exon 1 (Figure 1A) and C with T at -466 (Figure 1B) and T with G at -215 (Figure 1C) in intron 1 of the *MDM2* gene. Nucleotide C was inserted at positions -824 to -818 (C-to-C insertion) of the *p53* gene (Figure 2). We identified 9 genotypes related to *MDM2* and *p53* polymorphisms containing nucleotide alterations (I-IX; Table 2); genotypes I, II, III, VI, and VIII were observed in 1 individual each, types V and IX were observed in 2 individuals each, and types IV and VII were observed in 4 individuals each. Promoter nucleotides of the *p16*^*INK4a*^ gene at positions -735, -493, and -191 were examined using samples obtained from 9 individuals randomly selected from the 17 Japanese individuals. We observed substitution of nucleotide A with nucleotide G in *p16*^*INK4a*^ at position -191 (Figure 3); no nucleotide substitutions were observed at positions -735 and -493. This nucleotide alteration was observed in 3 individuals who were homozygous A/A, 4 individuals who were heterozygous A/G, and 2 individuals who were homozygous G/G at -191.

**Figure 4:**
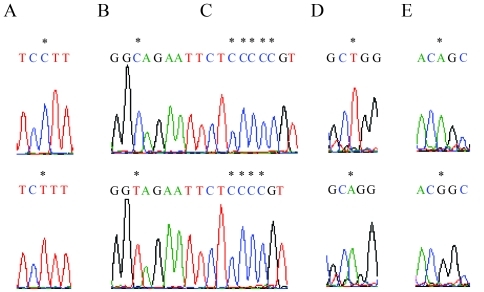
Nucleotides in the vector constructs of *p16*^*INK4a*^ at positions -1602, -871, -862 to -858, -315, and -191. (A-E) Genomic DNA was amplified by PCR with a forward primer at positions -1703 to -1683 and a reverse primer at -93 to -113 of the *p16*^*INK4a*^ gene and then cloned into PGVB2 vectors.  The nucleotides of the cloned sequences were examined using primers at positions -1706 to -1684, -950 to -931, -381 to -361, and -248 to -228. (*) shows nucleotides at positions -1602 (A), -871 (B), -862 to -858 (C), -315 (D), and -191 (E) of the *p16*^*INK4a*^ promoters.  Nucleotides around these polymorphic positions (*) are indicated.

The polymorphic *MDM2*, *p53*, and *p16*^*INK4a*^ sequences were cloned into PGV-B2 vectors, and 3 constructs of MDM2 PGV-ACT, PGV-ACG, and PGV-GTT (Table 3), 2 constructs of p53 PGV-7C and PGV-8C (Table 4), and 2 constructs of p16^INK4a^ PGV-A (containing nucleotide A at -191) and PGV-G (containing nucleotide G at -191) were obtained. Each of the cloned sequences was consistent with the nucleotide sequences determined by directly sequencing the PCR products (Figure 4E and other data not shown). In confirmation of the cloned sequences of *p16*^*INK4a*^, we also observed 2 alleles of *p16*^*INK4a*^ containing a substitution of nucleotide C with T at position -1602 (Figure 4A), and substitutions of C with T at -871 (Figure 4B), T with A at -315 (Figure 4D), and A with G at -191 (Figure 4E) and deletion of nucleotide C at positions -862 to -858 (Figure 4C). These nucleotide alterations at positions -1602, -871, -862 to -858, and -315 were ascertained by direct sequencing of PCR products (Figure S1). The *MDM2* nucleotides at positions -339, -333, and -218 and positions -237 to -234, -231 to -228, and -223 to -221 (G-to-G insertion) and the *p53* nucleotides at positions -512 to -511 (G-to-G deletion) and -479 to -477 (T-to-T insertion) differed from the nucleotides at the corresponding positions in the *MDM2* and *p53* sequences deposited in the GenBank database (accession numbers U39736.1, U28935.1, X54156.1, J04238.1, and M13111.1) (Tables 3  and 4). However, these nucleotides were identical among all study individuals (Tables 3  and 4). The *p53* nucleotides at positions -512 to -511 (G-to-G deletion) and -479 to -477 (T-to-T insertion) in the healthy individuals were consistent with the corresponding nucleotides in the *p53* sequence in the GenBank database (accession number U94788.1, Table 4).

###  Influence of promoter polymorphisms on reporter gene activity in different cell lines

**Figure 5:**
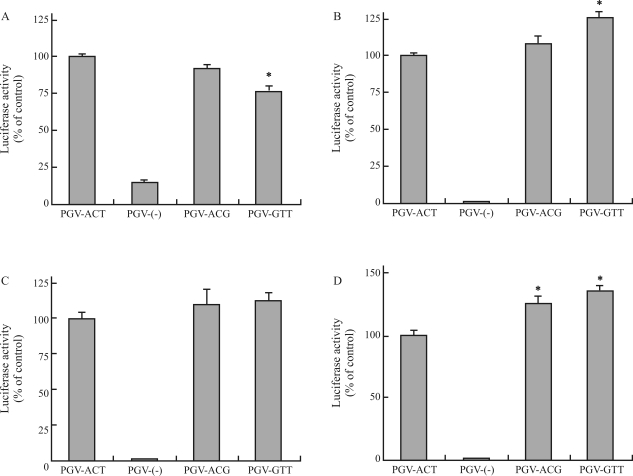
Effects of *MDM2* polymorphisms on luciferase activity in different cell lines. (A-D) Colo320DM (A), U251 (B), T98G (C), and YMB-1 (D) cells were transfected with the indicated vector constructs of PGV-(-), PGV-ACT, PGV-ACG, and PGV-GTT. The transfected cells were cultured for 24 h, and luciferase activity was determined. Data are shown as percentages of values obtained from the PGV-ACT transfectants and are expressed as the mean ± SEM (n = 8) (A-D). *: Significant difference compared with the PGV-ACT transfectant.

**Figure 6:**
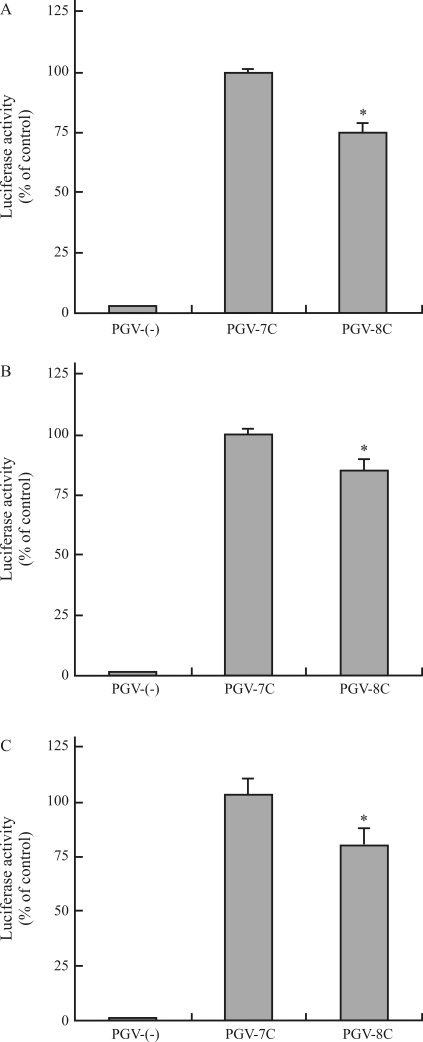
Effects of the *p53* poly(C) polymorphism on luciferase activity in different cell lines. (A-C) Colo320DM (A), U251 (B), and T98G (C) cells were transfected with the indicated vector constructs of PGV-(-), PGV-7C, and PGV-8C. The transfected cells were cultured for 24 h, and luciferase activity was determined. Data are shown as percentages of values obtained from the PGV-7C transfectants and are expressed as the mean ± SEM (n = 8). *: Significant difference compared with the PGV-7C transfectant.

**Figure 7:**
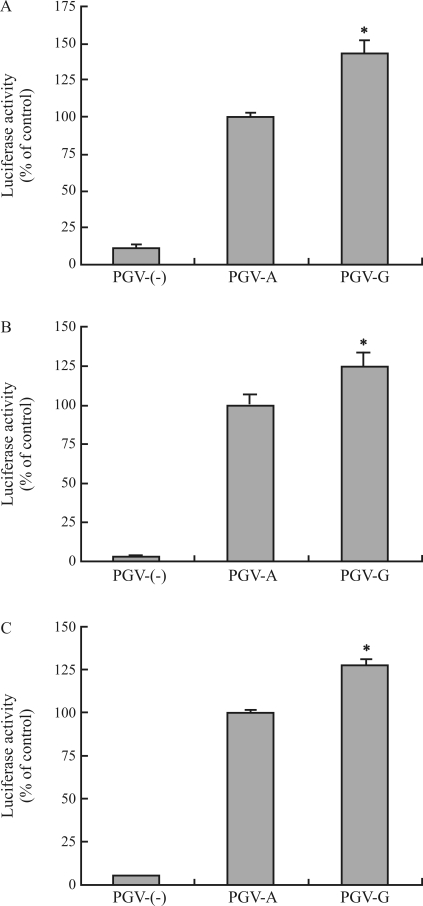
Effects of the *p16*^*INK4a*^ polymorphism at position -191 on luciferase activity in different cell lines. (A-C) Colo320DM (A), U251 (B), and T98G (C) cells were transfected with the indicated vector constructs of PGV-(-), PGV-A, and PGV-G. The transfected cells were cultured for 24 h, and luciferase activity was determined. Data are shown as percentages of values obtained from the PGV-A transfectants and are expressed as the mean ± SEM (n = 8). *: Significant difference compared with the PGV-A transfectant.

Several cell lines, including the colo320DM, U251, and T98G cell lines, were transfected with vector constructs containing *MDM2*, *p53*, and *p16*^*INK4a*^ polymorphisms, and the activity of the luciferase reporter gene in the transfected cells was examined to assess whether these polymorphisms alter gene promoter activity. The level of luciferase activity increased in cell lines transfected with the MDM2 construct PGV-ACT (containing nucleotides A at position -628, C at -466, and T at -215) (Figure 5); slight luciferase activity was detected in cell lines transfected with the empty vector PGVB2 (PGV-(-)). A significant decrease or increase was found in the luciferase activity of Colo320DM or U251 cells transfected with the MDM2 construct PGV-GTT (containing an A-to-G substitution at -628 and a C-to-T substitution at -466) compared to the activity in cells transfected with PGV-ACT (Figure 5A, B). Similar increase in luciferase activity was also observed in YMB-1 (Figure 5D) or HeLa (data not shown) cells transfected with the PGV-GTT construct. The MDM2 construct PGV-ACG (containing a T-to-G substitution at -215) did not significantly alter luciferase activity in the transfected Colo320DM and U251 cells (Figure 5A, B) and increased the activity in the transfected YMB-1 cells (Figure 5D). PGV-ACT, PGV-ACG, and PGV-GTT T98G transfectants had similar luciferase activities (Figure 5C). In the case of Colo320DM, U251, and T98G cells, the luciferase activities of cells transfected with p53 PGV-8C (containing nucleotide C insertion) and cells transfected with p16^INK4a^ PGV-G were significantly lower and higher than the activities of cells transfected with p53 PGV-7C and cells transfected with p16^INK4a^ PGV-A, respectively (Figures 6  and 7). The p53 PGV-8C and p16^INK4a^ PGV-G vectors did not decrease and increase the luciferase activity in the transfected YMB-1 and HeLa cells, respectively (data not shown).

## Discussion

In the present study, we found that normal Japanese individuals exhibit polymorphisms in the *MDM2*, *p53*, and *p16*^*INK4a*^ promoter regions (Figures 1, -4 and Figure S1) and that these individuals harbor several nucleotides in the *MDM2* and *p53* promoters that are different from those in the GenBank database (Tables 3  and 4); however, these nucleotides were identical among all the Japanese individuals included in this study. This result implies that the *MDM2* and *p53* promoter nucleotides differ among different populations, including individuals within the same population. The frequency of SNP309, a polymorphism involving nucleotide G, in intron 1 of the *MDM2* gene is low among African Americans and high in other populations (Dharel *et al.*, 2006; Hu *et al.*, 2006; Millikan *et al.*, 2006; Park *et al.*, 2006; Atwal *et al.*, 2007), including Ashkenazi Jewish, White, and Japanese populations. The polymorphism that we identified at position -215 in the intronic *MDM2* promoter was polymorphism SNP309 of *MDM2*. The G allele frequency of SNP309 at -215 in our Japanese samples was high (44.1%; 15 of 34 alleles) and similar to that in healthy individuals who visited a Japanese hospital (54%; 52 of 96 alleles) (Dharel *et al.*, 2006). African Americans, Caucasians, and Ashkenazi Jewish populations have other *MDM2* polymorphisms involving nucleotide substitutions from A to G (rs937283) and C to T (rs2870820), which are registered in the dbSNP database, at rates of 27%, 34%, and 25% and of 6%, 34%, and 0%, respectively (Atwal *et al.*, 2007). These polymorphisms are located at position -628 in exon 1 (rs937283) and position -466 in intron 1 (rs2870820) of the *MDM2* gene and corresponded to the nucleotides observed in our samples. The frequency of the G or T allele in healthy Japanese individuals was 29.4% each (10 of 34 alleles). The T allele frequency in Japanese individuals (29.4%) was higher than that in African American (6%) and Ashkenazi Jewish (0%) populations. Families with Li-Fraumeni (LF; an autosomal dominant cancer-predisposition syndrome) and LF-like families exhibit a nucleotide deletion in the *p53* promoter (Attwooll *et al.*, 2002), and mutations have been identified at 15 positions in the *p53* promoter in Taiwanese patients with uterine leiomyoma (Hsieh *et al.*, 2007). The nucleotide deletion (position -342) and 4 mutations (positions -250, -216, -103, and -33) within 15 positions have also been observed in control subjects at rates of 0.3% (Attwooll *et al.*, 2002) and 1.8%-4% (Hsieh *et al.*, 2007), respectively. These polymorphisms were not observed in our healthy Japanese samples, and the poly(C) polymorphism of *p53* at positions -824 to -818 (C-to-C insertion) found in our samples was observed at a high frequency of 41.2% (14 of 34 alleles). It has been reported that the nucleotides at positions -735, -493, and -191 of *p16*^*INK4a*^ are substituted not only in melanoma families in the United Kingdom, the United States, Italy, and Australia but also frequently in control populations (Harland *et al.*, 2000). In these control populations, the frequencies of homozygous A/A, heterozygous A/G, and homozygous G/G polymorphisms at position -191 of *p16*^*INK4a*^ are 38%, 48%, and 14%, respectively (Harland *et al.*, 2000). The corresponding frequencies of these polymorphisms in our samples were 33.3%, 44.4%, and 22.2%. In addition, the frequency of polymorphisms C/C, C/C, 5C/5C, and T/T at positions -1602, -871, -862 to -858, and -315 of *p16*^*INK4a*^ was 44.4%; that of C/C, C/T, 5C/4C, and T/A was 22.2%; that of C/C, T/T, 4C/4C, and A/A was 22.2%; and that of C/T, C/C, 5C/5C, and T/T was 11%. Thus, the promoter polymorphisms in *MDM2* at positions -215, -628, and -466, in *p53* at positions -342, -250, -216, -103, and -33, and in *p16*^*INK4a*^ at positions -735, -493, and -191 were differentially distributed in some normal and control populations. In this study, polymorphisms were observed among healthy Japanese individuals in the sequence of *MDM2* at positions -628 and -466, of *p53* at positions -824 to -818, and of *p16*^*INK4a*^ at positions -1602, -871, -862 to -858, -315, and -191.

The promoter region of *MDM2* includes multiple transcription-factor response elements (Phelps *et al.*, 2003; Bond *et al.*, 2004), including the p53-responsive element (positions -93 to -74 and -55 to -36) (Zauberman *et al.*, 1995). Reporter gene activity is increased in wild-type p53-expressing HeLa cells transfected with the G allele of *MDM2* SNP309 (Bond *et al.*, 2004); the polymorphism SNP309 is located adjacent to a binding site of the transcription factor SP1 in the *MDM2* promoter and can alter SP1 binding at this site. The *MDM2* G allele of SNP309 observed at -215 similarly increased reporter activity in HeLa cells (data not shown) and YMB-1 cells (Figure 5D). In contrast, the reporter luciferase activity of Colo320DM, U251, and T98G cells transfected with SNP309 at -215 was not altered (Figure 5A, B, C). We also observed a decrease, increase, or no change in luciferase activity in transfected Colo320DM, T98G, U251, HeLa, or YMB-1 cells that harbored the *MDM2* polymorphism at both positions -628 and -466 (Figure 5). Deletion of a promoter region of *MDM2* at positions -725 to -311, which include the -628 and -466 polymorphic positions, did not alter luciferase activity in Colo320DM cells and increased luciferase activity in other cells (Figure S2). This deletion experiment indicated that luciferase activity in Colo320DM cells may not be regulated constitutively by transcription factors through the *MDM2* promoter sequence containing the polymorphic sites -628 and -466 and that luciferase activity in other cells appears to be negatively regulated by these factors via the promoter containing the polymorphic sites. The reporter gene activity induced by polymorphism is thought to be caused by cell type-dependent regulation of the promoter machinery. A search for transcription factors that can bind to sequences around the polymorphic sites was carried out in the TRANSFAC database by using the MatInspector program (Wingender *et al.*, 2000). Database analysis showed that the -466 *MDM2* polymorphism affected the matrix similarity of the transcription factor MZF1. The *MDM2* polymorphism at both positions -628 and -466 may alter promoter activity via a known regulatory factor at position -466.

Patients with diseases such as cancer show not only nucleotide deletions and mutations (Pollock *et al.*, 2001; Attwooll *et al.*, 2002; Taniai *et al.*, 2002; Fombonne *et al.*, 2005; Hsieh *et al.*, 2007) but also nucleotide methylation (Herman *et al.*, 1996; Narimatsu *et al.*, 2004; Amatya *et al.*, 2005) in the promoter regions of *p53* and *p16*^*INK4a*^. The deletion at positions -346 to -338 (C/EBP site) of *p53* in LF (Attwooll *et al.*, 2002) and the mutation at position -250 of *p53* in patients with uterine leiomyoma (Hsieh *et al.*, 2007) are located within CpG dinucleotides. The locus of the *p53* poly(C) polymorphism at positions -824 to -818 was adjacent to CpG sequences, which are located at positions -826 to -825 and -818 to -817, and the -191 nucleotide G of *p16*^*INK4a*^ was located within CpG sites. Methylation of the *p16*^*INK4a*^ promoter has been found in noncancerous tissues in 3 of 21 (14%) individuals with non-cirrhotic virus-positive livers (Narimatsu *et al.*, 2004). Exposure to arsenic differentially stimulates the methylation of *p53* and *p16*^*INK4a*^ promoters (Chanda *et al.*, 2006). We did not observe nucleotide alterations in these methylated nucleotides in the *p53* and *p16*^*INK4a*^ promoters among normal Japanese individuals.

Some transcription factors interact with *p53* and *p16*^*INK4a*^ promoters and regulate gene promoter activity through these promoter regions (Furlong *et al.*, 1996; Kirch *et al.*, 1999; Myöhänen and Baylin, 2001; Xue *et al.*, 2004; Wu *et al.*, 2007). The polymorphic sites identified in these promoters in the present study were not located within their transcription factor-binding sites. Promoter methylation of *p53* is induced in the T98G cell line, and expression of the *p53* gene is upregulated by treatment of the cell line with 5-aza-2'-deoxycytidine, a methylation inhibitor, (Amatya *et al.*, 2005). Luciferase reporter gene activity was altered in T98G cells harboring the *p53* poly(C) polymorphism (Figure 6C). Chromatin immunoprecipitation with an antibody against the methyl-CpG binding domain protein (MBD) 2, an MBD family protein (Hendrich and Bird, 1998), revealed that the MBD2 complexes extracted from human colon cell lines bind to the *p16*^*INK4a*^ promoter region (from position -494 to -101) and that this binding is abolished by treatment with the methylation inhibitor 5-aza-2'-deoxycytidine (Magdinier and Wolffe, 2001). It was also revealed that although HeLa cells express MBD2 (Billard *et al.*, 2002), MBD2 does not bind to the *p16*^*INK4a*^ promoter region (Magdinier and Wolffe, 2001). Database analysis with the TFSEARCH program showed that several transcription factors, including those of SP1, CREB, and NF-κB, can bind to the *p53* poly(C) and *p16*^*INK4a*^ -191 sites and that nucleotide alterations at these sites affect the similarity of sequences of the substrates for the transcription factors activator protein 2 and ATF or NF-κB, etc., respectively. The regions around and within the *p53* poly(C) sequence are rich in guanine and cytidine nucleotides and are similar in part to the consensus sequences for transcription factors such as activating enhancer-binding protein 2 (Hilger-Eversheim *et al.*, 2000) whose gene includes the p53-responsive sequence (Li *et al.*, 2006). p53 can form complexes with SP1, CREB-binding protein, and NF-κB protein (Borellini and Glazer, 1993; Huang *et al.*, 2007). The *p53* poly(C) and *p16*^*INK4a*^ -191 polymorphisms did not decrease and increase luciferase activity in the YMB-1 and HeLa cells expressing wild-type p53, respectively. In contrast, these polymorphisms altered luciferase activity in the Colo320DM, U251, and T98G cells (Figures 6  and 7) that harbored p53 mutants at positions codon 248, 273, and 237, respectively. In each case, the polymorphism-induced reporter gene activity seems to be attributable to cellular differences in promoter regulation that occurs through the binding of DNA-interacting proteins; the regulation may result in a decrease or increase in reporter activity and may depend on cellular methylation of CpG nucleotides and on the ability of transcription factors.

Patients with cancers exhibit differential MDM2 and p53 protein expression in gliomas and breast cancers, which contain the G and/or T alleles of SNP309 (Tsuiki *et al.*, 2007; Krekac *et al.*, 2008), and differential p16^INK4a^ protein expression in gastric cancers in the absence of methylation (Tsujie *et al.*, 2000). Gene expression analysis performed using the TCGA database revealed that *MDM2* and *p16*^*INK4a*^ gene expression levels differ among patients with cancers, including those with glioblastoma multiforme; it is not known whether the regulatory regions of these genes have polymorphisms. Chemotherapeutic drugs increase the transcript level of p53 target genes in cells expressing wild-type MDM2, and the increased levels decrease in cells expressing the SNP309 G allele (Arva *et al.*, 2005). In addition, *MDM2* promoter activity is increased through the p53-binding site in the *MDM2* gene in cells expressing the thyroid hormone receptor (Qi *et al.*, 1999) and through multiple transcription-factor response elements in estrogen receptor (ER) α-positive breast cancer cells (Phelps *et al.*, 2003), and *p16*^*INK4a*^ reporter activity is increased in senescent cells in comparison with that in young cells (Wang *et al.*, 2001). Breast cancer YMB-1 and cervix epithelioid carcinoma HeLa cell lines are ERα-positive and -negative cells, respectively. The region around the *MDM2* polymorphic site at position -466 was a possible binding site of the transcription factor GATA (Phelps *et al.*, 2003) whose gene expression is increased in ERα-positive cells treated with estradiol (Eeckhoute *et al.*, 2007). The normal Japanese individuals who participated in our study included 13 male and 4 female students. Further studies are needed to clarify the significance of promoter polymorphisms in the variations in gene expression among individuals, in responses to chemical and hormone stimuli in individuals of different genders and ages, and under normal and aberrant conditions.

Research has been conducted on the construction of common patterns of sequence variations in the human genome (International HapMap Consortium, 2005). Polymorphism SNP309 is present in linkage disequilibrium with other *MDM2* intronic polymorphisms, and the linkage differs between different populations: Ashkenazi Jewish and Caucasian populations or African Americans (Atwal *et al.*, 2007). Exon 1 of the *p14*^*ARF*^ gene (Robertson and Jones, 1998) is located approximately 20 kb upstream of the first *p16*^*INK4a*^ exon 1 and splices into the common exons 2 and 3 shared with *p16*^*INK4a*^ in a different reading frame (Mao *et al.*, 1995). A Korean population has a promoter polymorphism of *p14*^*ARF*^ at position -1477, which is observed in strong linkage disequilibrium in a haplotype block, and the -1477 A allele of *p14*^*ARF*^ is associated with the methylation status of the *p14*^*ARF*^ promoter and alters the level of *p14*^*ARF*^ mRNA in colorectal tumor tissues (Kang *et al.*, 2008). Japanese populations frequently exhibited *p14*^*ARF*^ polymorphism at position -1477 (Figure S3). *p16*^*INK4a*^ polymorphisms at positions -871, -862 to -858, and -315 or *MDM2* polymorphisms at positions -628 and -466 appeared to be possible candidates for linkage disequilibrium. Single-nucleotide polymorphisms (SNPs) (Brookes, 1999) are the most frequently occurring sequence variation in a population and are associated with a predisposition to disease and susceptibility or tolerance to medication. They are useful as markers of individual constitution and as information for personalized medicine. A population-based study showed that the SNP309 polymorphism in *MDM2* and/or the common polymorphism at codon 72 in p53 are associated with predispositions to carcinomas in populations who visited Asian hospitals (Kawaguchi *et al.*, 2000; Hong *et al.*, 2005; Dharel *et al.*, 2006). Other studies showed that the *MDM2* SNP309 polymorphism or the p53 common polymorphism at codon 72 is not associated with or only weakly associated with predisposition to carcinomas in other populations (Millikan *et al.*, 2006; Krekac *et al.*, 2008; Tornesello *et al.*, 2009; Zubor *et al.*, 2009). The *p16*^*INK4a*^ -191 polymorphism does not segregate with disease in melanoma kindreds in the United Kingdom (Harland *et al.*, 2000). It is of interest to determine whether the promoter polymorphisms of *MDM2*, *p53*, and *p16*^*INK4a*^ identified in this study are present in linkage disequilibrium in a haplotype block and influence predisposition to diseases; our findings provide information for studies on the construction of common polymorphism patterns and for studies on the frequency distribution among different populations, including diseased patients. In the current study, we identified promoter polymorphisms in healthy Japanese individuals and found that gene promoter activity was altered by these polymorphisms in different cell lines. Our results indicate that promoter sequences of the *MDM2*, *p53*, and *p16*^*INK4a*^ genes differ among normal Japanese individuals.

## Supplementary Material

The following online material is available for this article:

Figure S1 Promoter polymorphisms of *p16*^*INK4a*^ at positions -1602, -871, -862 to -858, and -315.

Figure S2 Effects of the deletion of a region in the *MDM2* promoter on luciferase activity in Colo320DM, U251, T98G, YMB-1, and HeLa cells.

Figure S3 A promoter polymorphism of *p14*^*ARF*^ at position -1477 in normal Japanese individuals.

This material is available as part of the online article from http://www.scielo.br/gmb.

## Figures and Tables

**Table 1:** Primers used for PCR and sequencing

Primer position	Oligonucleotide primer
*MDM2* gene	
-725 to -704	5'-TCTGACCGAGATCCTGCTGCTT-3'
-465 to -442	5'-TCTATCGCTGGTTCCCAGCCTCTG-3'
-310 to -289	5'-TTCGGACGGCTCTCGCGGCGGT-3'
+96 to +75	5'-AAGCTACAAGCAAGTCGGTGCT-3'
-725 to -702	5'-TATTGGTACCTCTGACCGAGATCCTGCTGCTTTC-3'
+99 to +76	5'-TAGTAGATCTCTAAAGCTACAAGCAAGTCGGTGC-3'
*p53* gene	
-918 to -899	5'-GCTGGGAGTTGTAGTCTGAA-3'
-669 to -690	5'-CATTGTTGTATTCCTGAGTGCC-3'
-584 to -564	5'-GTGATAAGGGTTGTGAAGGAG-3'
-518 to -495	5'-GGGTGTGGATATTACGGAAAGCCT-3'
-233 to -210	5'-ACTTGCCCTTACTTGTCATGGCGA-3'
-221 to -240	5'-AGTAAGGGCAAGTAATCCGC-3'
+184 to +161	5'-AGGTCTCCCAACAATGCAACTCCT-3'
-920 to -898	5'-TACTGGTACCCTGCTGGGAGTTGTAGTCTGAAC-3'
+184 to +161	5'-TAGTAGATCTAGGTCTCCCAACAATGCAACTCCT-3'
*p16*^*INK4a*^ gene	
-2028 to -2009	5'-TACCTCCTTGCGCTTGTTAT-3'
-1706 to -1684	5'- ATGTTGGTCAGGCTTGTCTCGAA-3'
-1173 to -1192	5'-TGCCACACATCCTAAGCTAA-3'
-1198 to -1174	5'-CAGGTATTAGCTTAGGATGTGTGGC-3'
-950 to -931	5'-CTGGTCTAGGAATTATGACT-3'
-485 to -465	5'-TGTATCGCGGAGGAAGGAAAC-3'
-475 to -498	5'-TCCGCGATACAACCTTCCTAACTG-3'
-381 to -361	5'-AGGGAGGCCGGAGGGCGGTGT-3'
-248 to -228	5'-TGCCACATTCGCTAAGTGCT-3'
+214 to +194	5'-CTGCAAACTTCGTCCTCCAGA-3'
-1703 to -1683	5'-ATAGGTACCTTGGTCAGGCTTGTCTCGAAC-3'
-93 to -113	5'-CATAGATCTTCCTCTTTCTTCCTCCGGTGC-3'
PGV-B2 vector	
-59 to -40	5'-CTAGCAAAATAGGCTGTCCC-3'
+112 to +91	5'-CTTTATGTTTTTGGCGTCTTCC-3'

Nucleotide positions have been numbered by considering the positions of nucleotide C at the 5' end of exon 2 (Zauberman *et al.*, 1995) in the *MDM2* gene (accession number: U39736.1), nucleotide G (accession number: X54156.1) in the *p53* gene (Tuck and Crawford, 1989), nucleotide A at the initiation site for translation in the *p16*^*INK4a*^ gene (Hara *et al.*, 1996), and nucleotide G at the *KpnI* site of the PGV-B2 vector (sequence identical to that of pGL3-basic vector; GenBank accession number, U47295) as +1. The underlines indicate the recognition sites of the *KpnI* (singlet) and *BglII* (doublet) restriction enzymes.

**Table 2:** *MDM2* and *p53* promoter polymorphisms in normal individuals.

	Polymorphic position in the promoter regions of genes			
	*MDM2* gene			*p53* gene
Individual genotype	-628	-466	-215	-824 to -818
I	A/A	C/C	T/T	7C/8C
II	A/A	C/C	T/G	7C/7C
III	A/A	C/C	T/G	7C/8C
IV	A/A	C/C	G/G	7C/8C
V	A/A	C/C	G/G	8C/8C
VI	A/G	C/T	T/T	7C/7C
VII	A/G	C/T	T/T	7C/8C
VIII	A/G	C/T	T/G	7C/7C
IX	G/G	T/T	T/T	7C/7C

**Table 3:** *MDM2* promoter sequences in vector constructs.

	Position from the 5' end of exon 2 in the *MDM2* gene									
Type	-628	-466	-413	-339	-333	-237 to -234	-231 to -228	-223 to -221	-218	-215
U39736.1	A	C	N	A	C	4G	4G	3G	G	T
U28935.1	-	-	-	-	-	-	-	3G	C	G
PGV-ACT	A	C	C	G	G	5G	5G	4G	C	T
PGV-ACG	A	C	C	G	G	5G	5G	4G	C	G
PGV-GTT	G	T	C	G	G	5G	5G	4G	C	T

*MDM2* promoter regions were ligated to PGV-B2 vectors, and the ligated nucleotides were examined using primers at positions -725 to -704, -465 to -442, and -310 to -289. U39736.1 and U28935.1 represent sequences obtained from GenBank.

**Table 4:** *p53* promoter sequences in vector constructs.

	Position from nucleotide G in the *p53* gene		
Type	-824 to -818	-512 to -511	-479 to -477
X54156.1	7C	GG	3T
J04238.1	-	GG	3T
M13111.1	-	GG	3T
U94788.1	7C	G	4T
PGV-7C	7C	G	4T
PGV-8C	8C	G	4T

*p53* promoter regions were ligated to PGV-B2 vectors, and the ligated nucleotides were examined using primers at positions -918 to -899, -584 to -564, and -518 to -495. X54156.1, J04238.1, M13111.1, and U94788.1 are sequences obtained from GenBank.
