# Supplementary material for: Polymorphisms in promoter sequences of MDM2, p53, and p16INK4a genes in normal Japanese individuals
Source: Genet Mol Biol. 2010 Dec 1;33(4):615–26. doi: 10.1590/s1415-47572010000400004 (PMC3036159; doi:10.1590/s1415-47572010000400004)
Supplement: Figure S1 — Promoter polymorphisms of p16INK4a at positions -1602, -871, -862 to -858, and -315. [file gmb-33-4-615-suppl1.pdf]

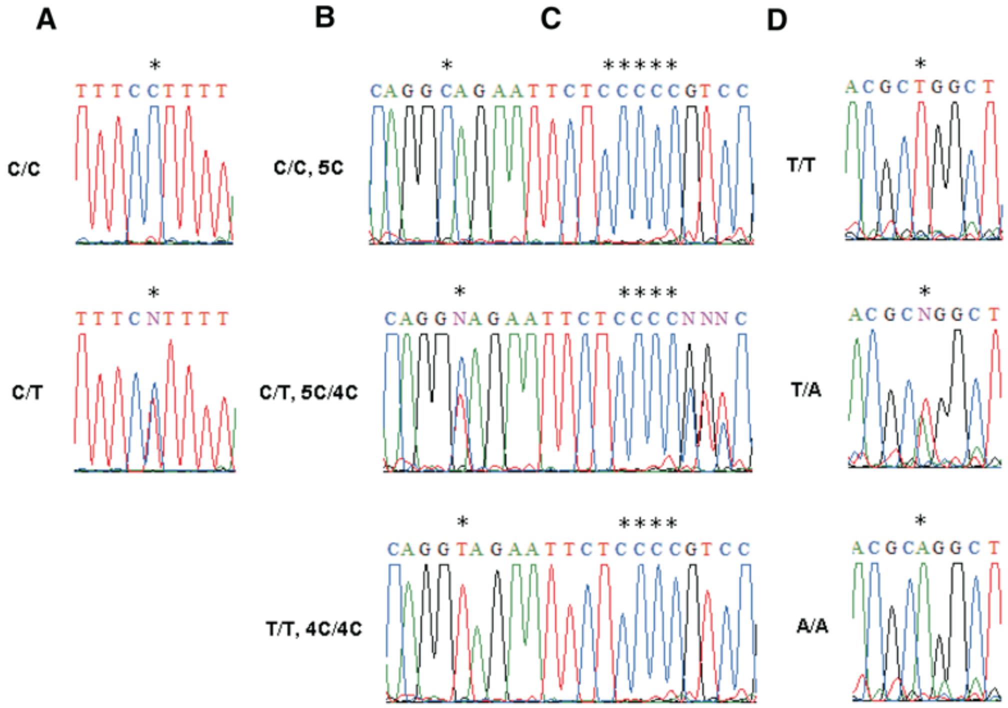

Figure S1 - Promoter polymorphisms of *p16*^{INK4a} at positions -1602, -871, -862 to -858, and -315. (A-D) Genomic DNA was amplified by PCR with forward primers at positions -2028 to -2009, -1198 to -1174, and -485 to -465 and reverse primers at -1173 to -1192, -475 to -498, and +214 to +194 of the *p16*^{INK4a} gene; nucleotide sequences were determined by directly sequencing the PCR products. These sequencing reactions were performed using primers at positions -1706 to -1684, -950 to -931, and -381 to -361. (*) shows nucleotides at positions -1602 (A), -871 (B), -862 to -858 (C), and -315 (D) in the *p16*^{INK4a} promoters, where "N" indicates heterozygous nucleotides. "NNNC" in (C) indicates sequences including "CGTC" and "GTCC," which result from nucleotide C deletion. Nucleotides around these positions (*) are indicated.
