# Supplementary material for: Polymorphisms in promoter sequences of MDM2, p53, and p16INK4a genes in normal Japanese individuals
Source: Genet Mol Biol. 2010 Dec 1;33(4):615–26. doi: 10.1590/s1415-47572010000400004 (PMC3036159; doi:10.1590/s1415-47572010000400004)
Supplement: Figure S2 — Effects of the deletion of a region in the MDM2 promoter on luciferase activity in Colo320DM, U251, T98G, YMB-1, and HeLa cells. [file gmb-33-4-615-suppl2.pdf]

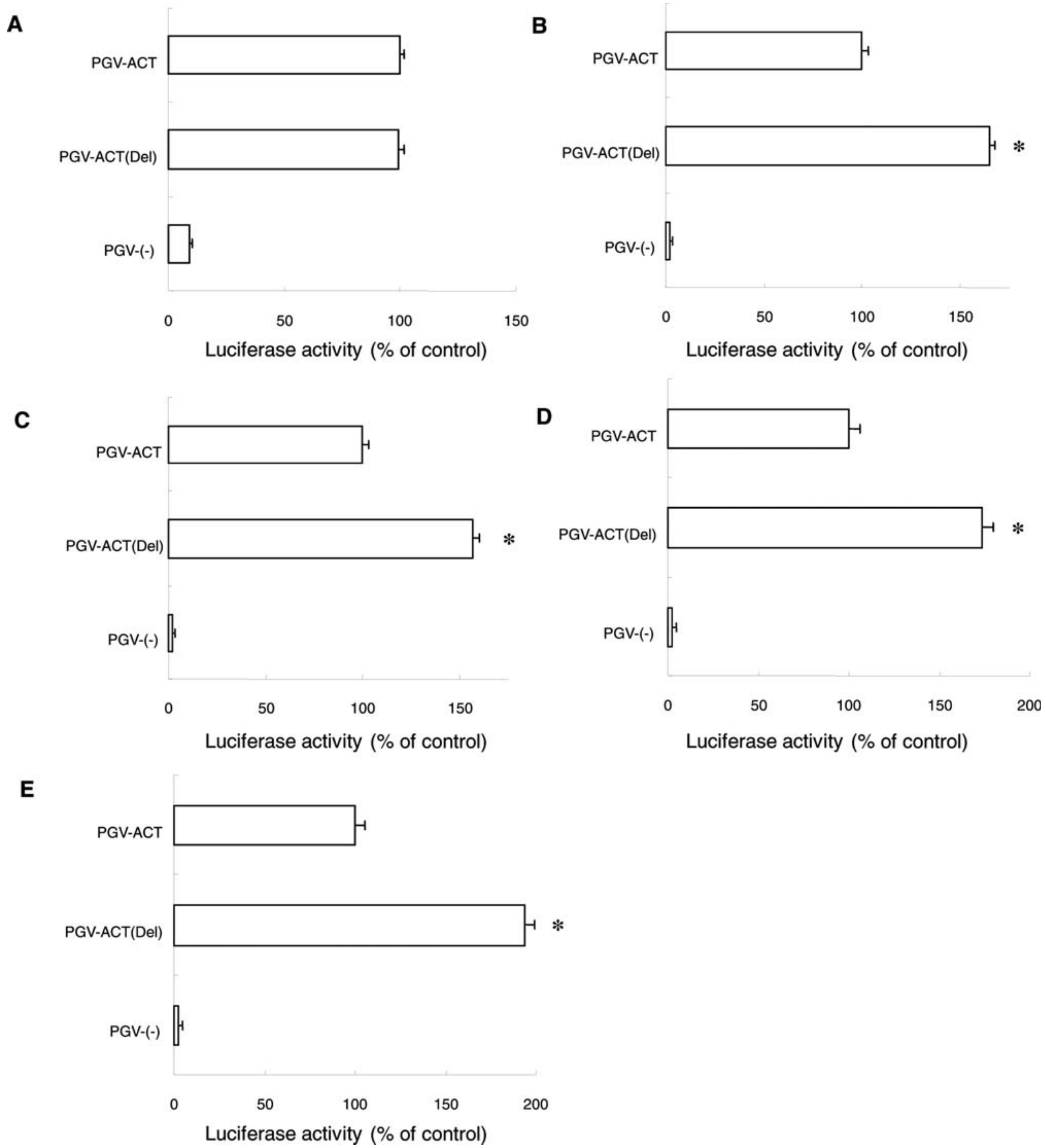

Figure S2 - Effects of the deletion of a region in the *MDM2* promoter on luciferase activity in Colo320DM, U251, T98G, YMB-1, and HeLa cells. Colo320DM (A), U251 (B), T98G (C), YMB-1 (D), and HeLa (E) cells were transfected with the indicated vector constructs of PGV-ACT (containing nucleotide A at position -628, C at -466, and T at -215), PGV-ACT(Del) (nucleotides at -725 to -311 deleted), and PGV-(-). The transfected cells were cultured for 24 h, and luciferase activity was determined. Data are shown as percentages of values obtained from the PGV-ACT transfectants and are expressed as the mean ± SD (n = 3). *: Significant difference compared with the PGV-ACT transfectant.
