# Supplementary material for: Polymorphisms in promoter sequences of MDM2, p53, and p16INK4a genes in normal Japanese individuals
Source: Genet Mol Biol. 2010 Dec 1;33(4):615–26. doi: 10.1590/s1415-47572010000400004 (PMC3036159; doi:10.1590/s1415-47572010000400004)
Supplement: Figure S3 — A promoter polymorphism of p14ARF at position -1477 in normal Japanese individuals. [file gmb-33-4-615-suppl3.pdf]

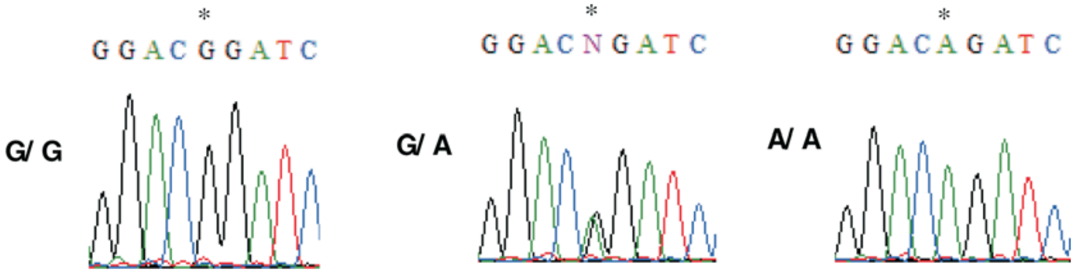

Figure S3 - A promoter polymorphism of *p14*^{ARF} at position -1477 in normal Japanese individuals. Genomic DNA was amplified by PCR with a forward primer at positions -1649 to -1627 and a reverse primer at -1090 to -1112 of the *p14*^{ARF} gene; the nucleotide sequences were determined by directly sequencing the PCR products. These sequencing reactions were performed using a primer at position -1649 to -1627. (*) shows nucleotides at position -1477 in the *p14*^{ARF} promoters, where "N" indicates heterozygous nucleotides containing G and A. Nucleotides around the polymorphic position (*) are indicated. The -1477 polymorphic frequency in 9 healthy Japanese individuals was 33.3% for homozygous G/G, 44.4% for heterozygous G/A, and 22.2% for homozygous A/A. Nucleotide positions are numbered with respect to nucleotide G (Robertson and Jones, 1998) at the initiation site of transcription in the *p14*^{ARF} gene (accession number: AF082338).
